# A Multiyear Model of Influenza Vaccination in the United States

**DOI:** 10.3390/ijerph14080849

**Published:** 2017-07-28

**Authors:** Arnold Kamis, Yuji Zhang, Tamara Kamis

**Affiliations:** 1 Information Systems and Operations Management Department, Sawyer Business School, Suffolk University, Boston, MA 02108, USA; 2 Alivia Technology, Boston, MA 02114, USA; yzhang@aliviatechnology.com; 3 Lexington High School, Lexington, MA 02421, USA; tnk1234567@gmail.com

**Keywords:** adults, influenza vaccination, public health, communication, cognitive bias

## Abstract

Vaccinating adults against influenza remains a challenge in the United States. Using data from the Centers for Disease Control and Prevention, we present a model for predicting who receives influenza vaccination in the United States between 2012 and 2014, inclusive. The logistic regression model contains nine predictors: age, pneumococcal vaccination, time since last checkup, highest education level attained, employment, health care coverage, number of personal doctors, smoker status, and annual household income. The model, which classifies correctly 67 percent of the data in 2013, is consistent with models tested on the 2012 and 2014 datasets. Thus, we have a multiyear model to explain and predict influenza vaccination in the United States. The results indicate room for improvement in vaccination rates. We discuss how cognitive biases may underlie reluctance to obtain vaccination. We argue that targeted communications addressing cognitive biases could be useful for effective framing of vaccination messages, thus increasing the vaccination rate. Finally, we discuss limitations of the current study and questions for future research.

## 1. Introduction

Vaccine avoidance is a serious problem, but its severity is not yet recognized by many adults. Their refusal to vaccinate contributes to increased incidence of preventable diseases, such as measles, putting population health in danger [1,2]. Influenza, which the majority of adults survive without serious side effects, can result in hospitalization or death, particularly among the elderly [3].

The United States’ Centers for Disease Control and Prevention (CDC) describes the benefits of influenza vaccines for vulnerable populations, such as children, the elderly, the chronically ill, and pregnant women. Influenza vaccination reduces the risk of influenza-related hospitalization for children and the elderly, while preventing chronic disease-based hospitalization for people with health conditions such as diabetes or chronic lung disease. It can also help prevent cardiac events in people with cardiac disease. Influenza vaccination reduces the risk of respiratory infections for pregnant women, and may protect their infants through antibodies passed via breast milk. Finally, the CDC points out that influenza vaccination helps not only the recipient but also the community through herd immunity. 

The CDC recommends influenza vaccination for everyone 6 months of age and older. The only contraindications are allergies to the influenza vaccine, eggs, or any of its ingredients, and a Guillain-Barré Syndrome (GBS) occurrence in the six weeks prior to influenza vaccination. Those who experience only hives as an allergic reaction to eggs are recommended to nevertheless receive the vaccine. Individuals with non-life threatening allergic reactions other than hives should be vaccinated under medical supervision. GBS is a rare autoimmune disorder associated with rapid onset muscle weakness, and sometimes paralysis [3]. Individuals who have a recent history of GBS, who are also at high risk for influenza complications, should nevertheless receive the influenza vaccination in some cases. Considering the rarity of GBS and egg allergies in adults, as well as the sub-populations within these groups who should nevertheless receive influenza vaccination, the vast majority of adults should obtain it annually [3].

There are many contributing factors to the problem of failure to vaccinate. The common factors include the following: vaccine efficacy vs. the unpleasantness of the vaccination process or side-effects, the perceived adequacy of one’s natural immunity against viruses, the cost in time or money, or a distrust in the medical establishment or pharmaceutical companies [1]. Many people may be complacent about influenza, believing their good current health is a strong enough defense against viruses [4]. Some may not be persuadable, even with the prospect of a severe influenza season and a steady campaign of appeals to personal health, public health, and the desire to protect one’s family. Other people, conversely, will obtain a vaccination readily, with only one or two reminders from their doctor [5]. Finally, there are in-between people, those who need several reminders from various sources. 

A review of the literature on influenza vaccination determined the most common reasons given for (not) obtaining it. Descriptively, the top three reasons for seeking influenza vaccinations: self-protection, protection of family or community, and protecting the patients of health care workers. The top three reasons for declining influenza vaccination: perception that one is healthy and not at risk [6], skepticism regarding vaccine efficacy [7], and unpleasant side-effects. In sum, there are many factors contributing to the public’s beliefs, attitudes, intentions, and ultimately whether a vaccination is obtained.

Given so many plausible contributing factors, it would be useful to model rigorously the variety of factors contributing to the likelihood of receiving a seasonal influenza vaccination. To better communicate with the segment of the population who may be persuaded, we need to understand the variety of factors weighing on the decision to vaccinate, including demographics (education level, income level, age, and gender), behavior (nutrition, exercise, smoking, etc.), health care coverage, and current health status.

In this paper, we present a classification model about whether a person receives a flu vaccination. The data is from the CDC’s Behavioral Risk Factor Surveillance system (BRFSS) annual survey, a nationwide phone-based survey about health-related behaviors on adults [8]. The response variable is the binary variable Influenza Vaccination, answering the question “During the past 12 months, have you had either a flu shot or a flu vaccine?” We review our methods in Section 2 and show the results in Section 3. In Section 4, we discuss the results, some implications, possible communication strategies, and some ideas for further research. Section 5 concludes with a summary of the paper.

## 2. Methods

The BRFSS data is publically available online [8]. It is a health-related phone survey of adults (at least 18 years of age), and conducted in fifty states, the District of Columbia, and three U.S. territories. State health departments use in-house interviewers or contract with universities or telephone call centers to administer the surveys throughout the year. It is the largest continuous health survey in the world, and is offered in both English and Spanish. Participants are administered a standardized core questionnaire, as well as optional modules and state-specific questions. The survey is conducted using Random Digit Dialing on cell phones and land lines. 

We used three recent years’ data of BRFSS: 2012 to 2014. First, the 2013 dataset was used to build the model. Specifically, a logistic regression was developed on 60% of the data from 2013, validated on a holdout sample of 20% of the data, and tested on the final 20% of the data. This is consistent with standard data mining practices to avoid model overfitting [9]. The best 2013 model was tested further on the 2012 and 2014 BRFSS datasets to assess whether predictors of vaccination changed over time. All data (sub)sampling was random, and there was no evidence of significant collinearity among the nine predictors in any model. Ultimately, the model was stable during the 2012–2014 time period.

We initially gathered data from the 2013 BRFSS dataset (*n* = 491,773) and performed transformations to clean and standardize the data. Some non-Gaussian numerical variables were transformed to be more Gaussian with a mathematical function, e.g., logarithm. Almost all the variables had a small fraction of Don’t Know/Not Sure, Refused to Answer, or Missing/No Answer/Blank. In our model, we grouped those responses into one category: Missing. For the variable Pneumococcal Vaccination, the proportion of Don’t Know/Not Sure was almost 10%, so we left that category as is. Ultimately, out of 359 possible variables, using knowledge of the domain, 75 variables were deemed to be clean, complete, and usable for modeling, i.e., having significant variability. 

We conducted a standard logistic regression, starting with the initial set of 75 usable predictor variables, and the binary response variable Influenza Vaccination [10]. The response variable is nearly balanced, e.g., 46% Yes vs. 54% No in the 2013 data. The fitted model predicted True if the fitted probability exceeded 0.5 and False otherwise. Our methodology for variable selection was stepwise: Forward Selection (Conditional), maximizing adjusted R-Squared, until the final model was obtained. The final model of nine predictors maximized predictive power (adjusted R-Squared), contained only statistically significant predictors, and contained little collinearity (VIF < 5 for each predictor). The response variable and the nine predictors are found in Table 1.

## 3. Results

Our final model to predict Influenza Vaccination from the data consists of nine predictor variables: Age, Pneumococcal Vaccination, Time Since Last Checkup, Highest Education Level Attained, Employment, Health Care Coverage, Number of Personal Doctors, Smoking Status, and Annual Household Income. Table 2 shows the frequency statistics of all the categorical predictors in the 2012, 2013, and 2014 datasets.

**Figure 1:**
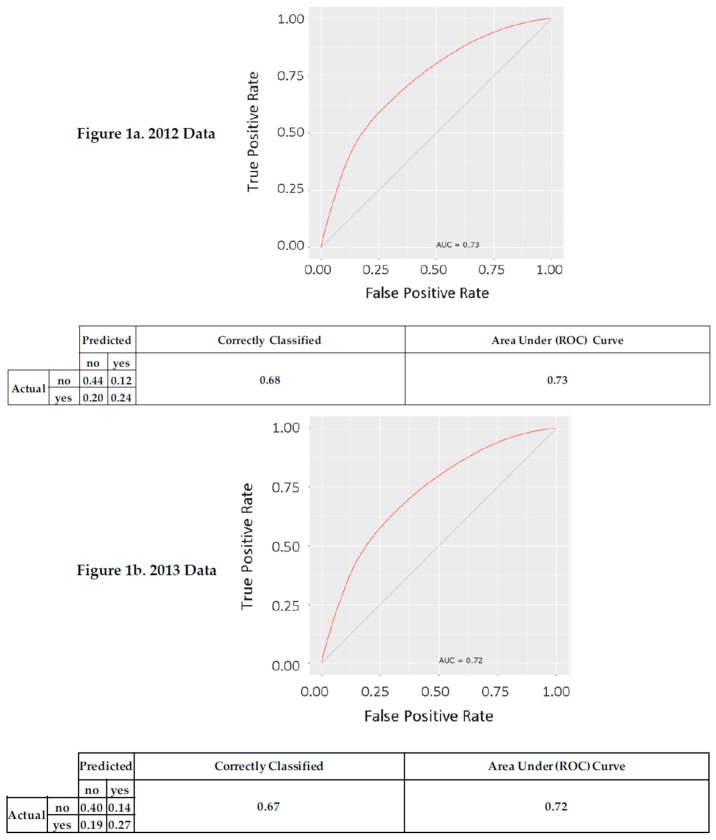

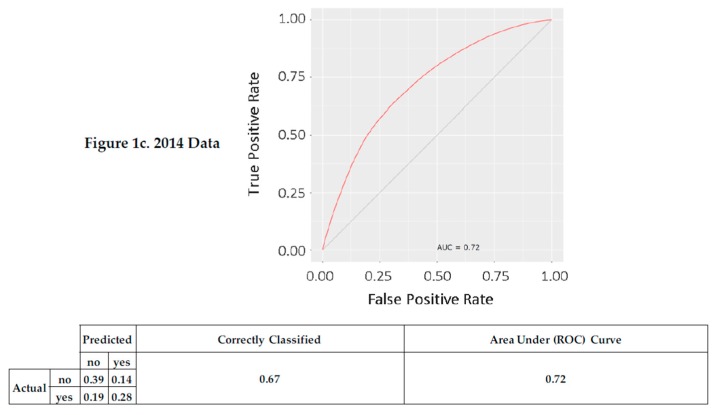
Confusion matrix and area under the receiver operating characteristic curve.

In Figure 1, the resulting Confusion Matrix and graph of Area Under the receiver operating Characteristic (AUC) curve show the predictive accuracy of the models. Figure 1b shows that sixty-seven percent of the cases were classified correctly as receiving influenza vaccination (27%) or not (40%), based on the nine predictors. The red line shows a smooth tradeoff between the false positive rate and the true positive rate. The gray diagonal line represents chance classification. The AUC for 2013 is 72% [12]. The same nine-predictor model applied to the 2012 and 2014 datasets yielded similar confusion matrices and AUC Curves (Figure 1a,c) [5,6]. The overall trend in vaccination is shown by the three confusion matrices. From 2012 to 2014, people receive an influenza vaccination at approximately the same rate, 44% (2012) to 46% (2013) to 47% (2014), whereas the model correctly classifies at a level of 67–68% across the three years. 

Overall, Table 3 shows the nine predictors, ranked by mean analysis of deviance, across the three years. This shows the relative importance of the nine predictors in a given year as well as relative importance from year to year.

We see stability among the predictors, with some variation in the rankings. Age, Pneumococcal Vaccination, and Time Since Last Checkup are the top three predictors, and they never change rank. The middle three predictors are Number of Personal Doctors, Employment, and Annual Household Income. The bottom three predictors are, Smoking Status, Health Care Coverage, and Highest Education Level Attained. Overall, the nine predictors constitute a stable model of influenza vaccination in the United States 2012–2014.

Table 4 shows the details of the logistic models for the three years. We consider each of the nine predictors and interpret the results. For each categorical variable, the referent category is the first one, which has a beta coefficient of zero in the table. We have omitted the Missing category. We did so because even as an aggregate category, the category’s proportion of the data for each variable was small, and the result would be less interesting to interpret.

*Age*: Age is the only numerical variable in the model. For every additional year of age, the odds ratio is 1.015, meaning the odds increase by 1.5% for having received an influenza vaccination.

*Pneumococcal Vaccination*: The referent category is Yes, received a pneumococcal vaccination. If the respondent instead receives no pneumococcal vaccination, the odds of an influenza vaccination decrease strongly with an odds ratio of 0.365, which means a decrease of 1 − 0.365 = 63.5% in the odds. If the respondent does not know/is not sure whether a pneumococcal vaccination was received, the odds ratio is 0.456 compared to the referent category.

*Time Since Last Checkup*: The referent category is having a checkup less than one year ago. The other categories show odds ratios (each compared to the referent case) steadily decreasing as the time since last checkup grows longer. If the last checkup was between 1 and 2 years ago, the odds decrease strongly compared to the referent case with an odds ratio of 0.716, which means a decrease of 1 − 0.716 = 28.4% in the odds. If the last checkup was between 2 and 5 years ago, the odds decrease further with an odds ratio of 0.579 compared to the referent case. If the last checkup was greater than 5 years ago, the odds decrease further (odds ratio = 0.452 compared to the referent case). The exception to this trend is the case of never having received a checkup, which is rare (< 1% of respondents). In this case, the odds ratio (0.637 compared to the referent case) is between that of a checkup 1–2 years ago and a checkup 2–5 years ago.

*Number of Personal Doctors*: The referent category is having exactly one personal doctor. If the respondent has more than one personal doctor, the difference is not statistically significant. If the respondent has no personal doctor, the odds of an influenza vaccination decrease strongly with an odds ratio of 0.657, which means a decrease of 34.3% over having exactly one personal doctor. 

*Annual Household Income*: The categories are ordinal, and income of $10,000 or less was used as the referent category. If the respondent has an annual household income of $10,000–$15,000, the difference is not statistically significant. Starting at the next bracket (category of income >$15,000), the odds of an influenza vaccination gradually increase as the income increases: from an odds ratio of 1.054 for the income level of $15,000–$20,000, up to an odds ratio of 1.447 for the income level of greater than $75,000 (all odds ratios are compared to the referent case; see Table 4 for all categories).

*Smoking Status*: The referent category is smoking Every Day. If the respondent only smokes Some Days, the odds of an influenza vaccination increase with an odds ratio of 1.134, which means an increase of 13.4% over smoking every day. If the respondent is a former smoker (ex-smoker), the odds of an influenza vaccination increase more strongly with an odds ratio of 1.416 compared to the referent case, which is an additional 28.2% over smoking only some days. If the respondent has never smoked, the odds increase is not as much as the former smokers, with an odds ratio of 1.372 compared to the referent case.

*Health Care Coverage*: The referent category is Yes, having some kind of health care coverage, private or public. If the respondent answers No, the odds of an influenza vaccination decrease strongly with an odds ratio of 0.635, which means a decrease of 36.5% over having some kind of coverage.

*Employment*: The referent category is Employed for Wages. If the respondent is Self-employed, the odds of an influenza vaccination decrease strongly with an odds ratio of 0.629 compared to the referent case, which means a decrease of 1 − 0.629 = 37.1% in the odds. If the respondent is out of work for more than 1 year, the odds decrease with an odds ratio of 0.847 compared to the referent case, which means a decrease of 1 − 0.847 = 15.3% over being Employed for Wages. The cases of being out of work for less than 1 year and being a homemaker show odds decrease quite similar to being out of work for more than 1 year, with odds ratios at 0.865 and 0.870, respectively, compared to the referent case. The only category more likely to have an influenza vaccination is Student. If that is the category, the odds increase slightly with an odds ratio of 1.077, which means an increase of 7.7% over being Employed for Wages. If the respondent is Retired, the odds decrease slightly with an odd ratio of 0.972, which means a decrease of 2.8% over being Employed for Wages. The category of Unable to Work is not statistically different from being Employed for Wages.

*Highest Level of Education Attained:* The referent category is Did Not Graduate High School. If the respondent is a high school graduate, the odds of an influenza vaccination decrease slightly with an odds ratio of 0.964 compared to the referent case, with a marginally significant *p*-value between 0.01 and 0.05. If the respondent has attended but not graduated from college or technical school, the difference is not statistically significant from the referent case. If the respondent is a College or Technical School Graduate, the odds increase strongly with an odds ratio of 1.275 compared to the referent case.

## 4. Discussion 

We revisit the nine predictors and interpret the results broadly.

*Age*: Our results show an overall increase in the odds of influenza vaccination as one ages. Elderly individuals have more medical problems, e.g., stroke, myocardial infarction, pneumonia, chronic respiratory tract infections; thus they see a doctor more frequently, thereby receiving messages that they are at greater risk of complications from influenza [13,14,15,16]. Note: regarding influenza between 2010 and 2013, 54–70% of hospitalizations and 71–85% of deaths occurred among adults aged ≥65 years, adding to the significance of doctor-patient communications [17]. The elderly are thus reminded, perhaps frequently, that an influenza vaccination is effective at reducing and preventing morbidity and mortality [18]. The age effect is stable from 2012 to 2014, inclusive.

*Pneumococcal Vaccination*: The Pneumococcal Vaccination effect is the second most statistically significant of the nine predictors and it correlates positively with influenza vaccination. This could indicate convenience, obtaining both vaccinations in the same medical/clinical session. It could also indicate the lack of needle-phobia [19]. Note: the CDC’s policy for adults is that it recommends pneumococcal vaccination for all adults 65 years or older, adults who smoke, and others with certain medical conditions. The *Pneumococcal Vaccination* effect is stable from 2012 to 2014, inclusive.

*Time Since Last Checkup*: Time since one’s last checkup is a strong and consistent predictor of influenza vaccination. In general, the more recent a checkup, the greater the odds of an influenza vaccination. This could be a result of the reminder frequency or a recency effect. The one exception is adults who have never had a checkup. An adult in that category is somewhat more likely to receive an influenza vaccination than someone who has gone more than five years without a checkup. The *Time Since Last Checkup* effect is stable from 2012 to 2014, inclusive. 

*Number of Personal Doctors*: Those with no personal doctor are less likely to receive influenza vaccination than those with one or more personal doctors. One does not need a personal doctor to obtain an influenza vaccination, however [20]. Many clinics, local government offices, and pharmacies provide the vaccination at little to no cost. This may indicate that someone without a personal doctor may be disinclined to seek medical attention or may be misinformed about the benefits of vaccination [21]. The effect is stable from 2012 to 2014, inclusive. 

*Annual Household Income*: The models show the trend that greater income corresponds to greater odds of influenza vaccination, particularly at the higher income levels. This can be interpreted simply, that greater annual household income may indicate greater educational attainment and thereby obtaining information from reliable sources [22]. The *Annual Household Income* effect is stable from 2012 to 2014, inclusive, in most of the categories.

*Smoking Status*: The models show that in general, the less one smokes, the more likely it is that an influenza vaccination is obtained. It is interesting to note the exception: that an ex-smoker is even more likely to obtain vaccination than someone who has never smoked. A possible explanation may be that someone who quits smoking has consciously achieved a difficult health goal, whereas someone who has never smoked has not achieved that difficult goal. The ex-smokers are thus taking greater deliberate care of themselves, whereas those who have never smoked may be complacent [4]. The *Smoking Status* effect is stable from 2012 to 2014, inclusive.

*Health Care Coverage*: Those with any form of Health Care Coverage, private or public, are much more likely to receive influenza vaccination. A straightforward explanation is that the cost of the vaccination would not be a concern for those having coverage. The Health Care Coverage effect is stable from 2012 to 2014, inclusive. 

*Employment*: The results in this variable can be interpreted from both an infection and a communication perspective. Employed for Wages, the most common category, is one in which adults are usually exposed to many people at work. There are therefore two possible factors that increase the odds of vaccination in those adults: verbal reminders (to vaccinate) and viral sources (if they fail to do so). All the other categories expose an adult to less of these two sources, except perhaps for the Student category. Students are usually exposed to even more reminders and viruses, when on a university campus, than someone working for wages, but the Student category is not statistically significant in 2012 or 2014. Nevertheless, this pattern may suggest that availability of information and social interaction/contagion, are important considerations. The *Employment* effect is stable from 2012 to 2014, inclusive, in most of the categories.

*Highest Level of Education Attained:* The categories of High School Graduate and College or Technical School Attendee both have odds ratios close to 1 and *p*-values larger than 0.01. It is reasonable to consider these two categories combined with the referent case, so that this variable is simplified to two levels: College or Technical School Graduate vs. lower level of education attained. It can be seen that college or technical school graduates are significantly more likely to receive influenza vaccination. The reasons may be that they are more receptive to medical advice, more information literate [23], or less swayed by misinformation found on the Internet [21]. The *Highest Level of Education Attained* effect is stable for College or Technical School Graduate from 2012 to 2014, inclusive.

### Implications and Future Research

We address some implications of our findings, as well as communication strategies based on cognitive bias, limitations of this research, and ideas for future vaccination research.

Our results contain several findings that are not surprising. One is more likely to receive an influenza vaccination if one is older, has health care coverage, or has more frequent checkups, etc. Some readily apparent implications are suggested. For example, health communications in physician offices and pharmacies, e.g., posters or pamphlets, need to be appropriate to the age of the people likely to encounter them. Also, they should be designed for a lower education level to reach those who are less likely to vaccinate [24]. Some implications are less readily apparent. For example, one could tailor communications differently to the various employment categories: those who are Employed for Wages, Students, Homemakers, Unemployed, Self-Employed, Retired, or Unable to Work. Adults in those categories are exposed to different physical and social environments. The overall idea is to immunize the overall population and thereby achieve herd immunity [25,26] by optimizing communications to different subpopulations of the herd. Future research could investigate the tailoring of messages to these categories, and more generally, to people claiming to be too busy to vaccinate [27] or who postpone it repeatedly [28].

This paper shows that graduates of college or technical school are more likely to receive influenza vaccination, but even they can fail to receive a vaccination. When uninformed or uncertain, people—whether highly educated or not—may rely on cognitive biases. Cognitive biases are mental rules-of-thumb, i.e., heuristics that help people to make adequate decisions with a low level of effort. They are not optimal, systematic, or complete in their information processing, but they are usually adequate. For example, there are the Availability Heuristic and the Bandwagon Effect [29]. 

*Availability Heuristic*. The CDC, under its 2016 message strategy, referred to influenza as a serious disease that may result in hospitalization and possibly death [3]. This is scientifically accurate, but many people are ignorant or skeptical about the true risk posed by influenza. This may be an example of the Availability Heuristic, the estimation of likelihood based on the retrievability of cases from an individual’s memory, which is influenced by social communications. The United States population is currently greater than 324 million, and the upper limit of influenza deaths 1976–2007 is 49,000 in one season; less than 0.001 percent of the population has died. People are therefore much more likely to not know a person who died from influenza than to know a person who has died from it. 

*Bandwagon Effect.* For another example of cognitive bias, if a friend, family member, or coworker says that an influenza vaccination is not efficacious, the Bandwagon Effect may result. The Bandwagon Effect compounds the problem of someone being misinformed/skeptical, because such a person may be surrounded by likeminded people. This would be herd immunity against messages about the importance of influenza vaccination. 

Rather than consider cognitive biases to be irrational shortcuts to systemic, logical thinking, or ignore them completely, they could be used as a basis for decision guidance toward normative decision-making. The research question would be how to identify the cognitive bias in the given situation, or given subpopulation, and how to nudge it toward the normative vaccination behavior. Future research could investigate different cognitive biases that may need to be anticipated in order to help public health communication campaigns, whether aimed broadly at the overall population or narrowly at different subpopulations. Which populations can be persuaded to reconsider and rethink their cognitive biases if sent well-framed messages? 

Although the BRFSS datasets have been shown to be reliable and valid [30], it is important to keep several limitations in mind. One, this study, although examining 2012–2014 datasets, is multiple-snapshot, cross-sectional research. Cross-sectional designs do not allow for causal inferences. Two, data self-reported in response to a telephone survey could be underreported or biased in some variables, especially in ones with social stigma, e.g., smoking or low household income [3,31]. Finally, institutionalized individuals, including those living in hospitals, nursing homes, or prisons are excluded. 

Despite these limitations, our large sample size, rigorous data sampling, and model development with multiple validation and test samples, gives us a stable, multiple-year model of those who participated. There will always be some bias in survey response data. We assumed that any such bias is of low magnitude, given the rigorous reputation of BRFSS [30], and not specific to any particular demographic or behavioral category. There also may be timing issues, such as people not having a checkup and therefore not receiving vaccination reminders, in the year after a mild influenza season. The stability of the model over three years, 2012–2014, helps to guard against such timing issues.

To make our model more predictive, new research questions could be investigated. What is the impact of a person’s health information source preference: doctor, online consumer health websites, or social networks [32,33]? How influential are the beliefs about the health of a person’s online or offline community? How influential is a person’s trust in information from the health care system, personal doctors, pharmaceutical companies, government, or social media? How significant a factor is a person’s anxiety about vaccination infection [34] or fear of hypodermic needles [19]?

## 5. Conclusions

An influenza-induced fever is common and usually lasts two to four days [3,34]. Millions of people fall ill to influenza every year in the United States and recover in a few days [15]. Almost everyone has experienced it and knows others who have suffered similarly [16]. This commonly experienced episode needs to be counterbalanced with scientifically correct and appropriately communicated messages about influenza vaccination risk and the importance of vaccination.

The Internet has enabled the distribution of medical information, empowering patients to treat themselves or at least ask their doctors better, more informed questions. The Internet has also been getting noisier. The empowered patient has increased access to opinion or myth (noise) in addition to scientific information from the medical establishment (signal) [35,36]. Access to a variety of “medical beliefs, scientific and nonscientific evidence, and emotionally arousing stories of other patients” is growing [21]. Patients are questioning the legitimacy and recommendations of scientific authorities, allowing unscientific views the same legitimacy and weight as scientific ones [7]. The movement to greater patient engagement and empowerment may be unintentionally creating greater distrust of traditional authorities [1].

This article offers a stable, multiple-year model for predicting which adults received influenza vaccination in the United States from 2012 to 2014, inclusive. We also present some practical implications of the results and how health communications could be targeted. Our model improves our understanding of influenza vaccination in the United States, and how we might intervene. Outside the United States, there may be other challenges, i.e., vaccine shortages causing the prioritization of at-risk individuals and health care workers over other groups. In such environments, the ecosystem of vaccine supply, as well as social and viral contagion, may differ.

## Figures and Tables

**Table 1:** Predictor variables.

Variable	Definition/Question Asked [11]
Influenza Vaccination (response)	During the past 12 months, have you had either a flu shot or a flu vaccine?
Age	[What is your] age in years?
Pneumococcal Vaccination	A pneumonia shot (or pneumococcal vaccine) is usually given only once or twice in a person’s lifetime and is different from the flu shot. Have you ever had a pneumonia shot?
Time Since Last Checkup	About how long has it been since you last visited a doctor for a routine checkup? [A routine checkup is a general physical exam, not an exam for a specific injury, illness, or condition.]
Highest Level of Education Attained	[What is your highest] level of education completed?
Employment	Are you currently Employed for wages, Self-employed…?
Health Care Coverage	Do you have any kind of health care coverage, including health insurance, prepaid plans such as Health Maintenance Organizations, or government plans such as Medicare, or Indian Health Service?
Number of Personal Doctors	Do you have one person you think of as your personal doctor or health care provider? (If “No” ask “Is there more than one or is there no person who you think of as your personal doctor or health care provider?”)
Smoking Status	Four-level smoker status: Everyday smoker, Someday smoker, Former smoker, Non-smoker
Annual Household Income	[What] is your annual household income from all sources…: (If respondent refuses at any income level, code “Refused”.)

**Table 2:** Frequency statistics.

		2012		2013		2014	
Variable	Category	Freq.	Perc.	Freq.	Perc.	Freq.	Perc.
Pneumococcal Vaccination	Yes	159,557	34.40	161,784	35.61	163,282	36.97
	No	261,728	56.43	245,512	54.03	239,089	54.13
	Don’t Know/Not Sure	40,018	8.63	44,318	9.75	37,165	8.41
	Refused	2543	0.55	2770	0.61	2184	0.49
Time Since Last Checkup	Within past year	341,897	71.87	356,253	72.44	340,587	73.30
	Within past 2 years	56,165	11.81	57,338	11.66	53,668	11.55
	Within past 5 years	33,603	7.06	33,659	6.84	30,764	6.62
	5 or more years ago	33,267	6.99	33,717	6.86	29,293	6.30
	Don’t know/Not sure	5677	1.19	5743	1.17	5601	1.21
	Never	4655	0.98	4523	0.92	4047	0.87
	Refused	422	0.09	540	0.11	702	0.15
Highest Level of Education Attained	Did not graduate High School	42,351	8.90	42,132	8.57	37,003	7.96
	Graduated High school	139,501	29.33	142,953	29.07	131,325	28.26
	Attended College/Technical School	128,404	26.99	134,242	27.30	125,635	27.04
	Graduated College/Technical School	163,510	34.37	170,173	34.60	166,972	35.93
	Don’t know/Not sure/Missing	1921	0.40	2273	0.46	3,729	0.80
Employment	Employed for wages	196,289	41.31	202,169	41.17	189,219	40.93
	Self-employed	38,863	8.18	39,853	8.11	39,102	8.46
	Out of work for more than 1 year	14,409	3.03	14,081	2.87	10,759	2.33
	Out of work for less than 1 year	12,312	2.59	12,241	2.49	9755	2.11
	A homemaker	31,587	6.65	31,654	6.45	29,028	6.28
	A student	12,225	2.57	12,664	2.58	11,021	2.38
	Retired	132,138	27.81	138,311	28.16	136,639	29.56
	Unable to work	35,260	7.42	37,419	7.62	34,021	7.36
	Refused	2053	0.43	2713	0.55	2,703	0.58
Health Care Coverage	Yes	419,328	88.15	434,627	88.38	425,198	91.51
	No	54,801	11.52	55,242	11.23	37,642	8.10
	Don‘t Know/Not Sure	932	0.20	1023	0.21	934	0.20
	Refused	624	0.13	881	0.18	890	0.19
Number of Personal Doctor(s)	1 Personal Doctor	365,483	76.83	369,084	75.05	354,623	76.32
	2+ Personal Doctors	38,854	8.17	41,306	8.40	37,199	8.01
	No Personal Doctor	69,855	14.69	79,587	16.18	70,891	15.26
	Don’t know/Not Sure	1088	0.23	1176	0.24	1271	0.27
	Refused	406	0.09	620	0.13	679	0.15
Smoking Status	Every Day	54,940	11.55	55,157	11.22	47,122	10.14
	Some Days	21,160	4.45	21,455	4.36	19,242	4.14
	Former Smoker	135,426	28.47	138,218	28.11	128,629	27.68
	Never	254,492	53.50	261,621	53.20	248,500	53.48
	Don’t know/Refused/Missing	9669	2.03	15,322	3.12	21,171	4.56
Annual Household Income	≤$10,000	25,237	5.31	25,411	5.18	21,199	4.60
	$10,000–$15,000	26,406	5.56	26,759	5.45	22,943	4.98
	$15,000–$20,000	34,081	7.18	34,885	7.11	30,511	6.63
	$20,000–$25,000	40,384	8.50	41,738	8.50	37,531	8.15
	$25,000–$35,000	47,352	9.97	48,863	9.95	44,315	9.62
	$35,000–$50,000	59,877	12.61	61,506	12.53	57,418	12.47
	$50,000–$75,000	63,951	13.47	65,238	13.29	62,175	13.50
	≥$75,000	111,654	23.51	115,982	23.63	117,176	25.45
	Don’t know/Not sure	33,034	6.96	34,946	7.12	31,622	6.87
	Refused	32,961	6.94	35,559	7.24	35,553	7.72
Influenza Vaccination	Yes	202,640	43.61	208,387	45.79	205,328	46.41
	No	258,088	55.55	242,939	53.38	233,769	52.84
	Don’t Know/Not Sure	1399	0.30	959	0.21	1158	0.26
	Refused	2519	0.54	2791	0.61	2179	0.49

**Table 3:** Analysis of deviance.

	2012 Logistic Regression Deviance	2012 Logistic Regression Deviance Rank	2013 Logistic Regression Deviance	2013 Logistic Regression Deviance Rank	2014 Logistic Regression Deviance	2014 Logistic Regression Deviance Rank	2012–2014 Logistic Regression Mean Deviance Rank
Age	17,739.3	1	16,378.7	1	15,230.7	1	1.0
Pneumococcal Vaccination	13,599.2	2	10,199.2	2	10,833.8	2	2.0
Time Since Last Checkup	8280.7	3	7738.6	3	7577.9	3	3.0
Number of Personal Doctors	1659.5	5	2014.3	4	1910.7	4	4.3
Employment	2244.6	4	1577.3	6	1749.8	5	5.0
Annual Household Income	1543.9	6	2013.3	5	1681.4	6	5.7
Smoking Status	680.7	8	785.3	7	772.3	8	7.7
Health Care Coverage	837.1	7	709.8	8	701	9	8.0
Highest Education Level Attained	616.5	9	707.6	9	773.2	7	8.3

**Table 4:** Logistic Regressions for 2012–2014.

		2012 Logistic Regression							2013 Logistic Regression							2014 Logistic Regression						
	Chi−Square *p*−Value:	0.000							0.000							0.000						
	Pseudo R−Square (Optimistic):	0.401							0.389							0.383						
		Estimate	Std. Error	Odds Ratio	Odds Ratio 95%	Z Value	Pr(>|z|)		Estimate	Std. Error	Odds Ratio	Odds Ratio 95%	Z Value	Pr(>|z|)		Estimate	Std. Error	Odds Ratio	Odds Ratio 95%	Z Value	Pr(>|z|)	
	(Intercept)	−0.562	0.034	0.570	(0.533, 0.610)	−16.432	<2 × 10^−16^	***	−0.613	0.036	0.542	(0.505, 0.581)	−17.269	<2 × 10^−16^	***	−0.478	0.036	0.620	(0.578, 0.666)	−13.229	<2 × 10^−16^	***
Age	years	0.014	0.000	1.014	(1.013, 1.014)	38.508	<2 × 10^−16^	***	0.015	0.000	1.015	(1.014, 1.016)	39.791	<2 × 10^−16^	***	0.013	0.000	1.013	(1.013, 1.014)	35.685	<2 × 10^−16^	***
Pneumococcal Vaccination	yes (ref. cat.)	0.000							0.000							0.000						
	no	−1.120	0.010	0.326	(0.320, 0.333)	−113.618	<2 × 10^−16^	***	−1.008	0.010	0.365	(0.358, 0.372)	−101.101	<2 × 10^−16^	***	−1.025	0.010	0.359	(0.352, 0.366)	−103.598	<2 × 10^−16^	***
	Don’t Know/Not Sure	−0.739	0.017	0.478	(0.463, 0.493)	−44.737	<2 × 10^−16^	***	−0.785	0.016	0.456	(0.442, 0.471)	−48.287	<2 × 10^−16^	***	−0.611	0.017	0.543	(0.525, 0.561)	−36.362	<2 × 10^−16^	***
Time Since Last Checkup	<1 year ago (ref. cat.)	0.000							0.000							0.000						
	1–2 years ago	−0.374	0.014	0.688	(0.670, 0.706)	−27.702	<2 × 10^−16^	***	−0.334	0.014	0.716	(0.697, 0.735)	−24.365	<2 × 10^−16^	***	−0.347	0.014	0.707	(0.688, 0.726)	−25.427	<2 × 10^−16^	***
	2–5 years ago	−0.573	0.018	0.564	(0.544, 0.585)	−30.987	<2 × 10^−16^	***	−0.547	0.019	0.579	(0.558, 0.600)	−29.125	<2 × 10^−16^	***	−0.580	0.019	0.560	(0.539, 0.581)	−30.627	<2 × 10^−16^	***
	5+ years ago	−0.761	0.021	0.467	(0.449, 0.487)	−36.933	<2 × 10^−16^	***	−0.794	0.021	0.452	(0.434, 0.471)	−37.771	<2 × 10^−16^	***	−0.765	0.021	0.465	(0.446, 0.485)	−35.590	<2 × 10^−16^	***
	Never	−0.626	0.050	0.535	(0.485, 0.589)	−12.622	<2 × 10^−16^	***	−0.451	0.050	0.637	(0.578, 0.702)	−9.064	<2 × 10^−16^	***	−0.626	0.054	0.535	(0.481, 0.594)	−11.701	<2 × 10^−16^	***
Highest Education Level Attained	<High School Grad. (ref. cat.)	0.000							0.000							0.000						
	High School Graduate	0.028	0.017	1.029	(0.995, 1.064)	1.668	0.0953	.	−0.037	0.018	0.964	(0.931, 0.998)	−2.080	0.0375	*	−0.061	0.018	0.941	(0.908, 0.974)	−3.409	0.0007	***
	College/Tech. Sch. Attendee	0.093	0.017	1.098	(1.061, 1.136)	5.342	<0.0001	***	0.029	0.018	1.030	(0.994, 1.067)	1.613	0.1068	n.s	0.003	0.018	1.003	(0.968, 1.040)	0.173	0.8625	n.s.
	College/Tech. Sch. Graduate	0.276	0.018	1.317	(1.272, 1.365)	15.371	<2 × 10^−16^	***	0.243	0.018	1.275	(1.229, 1.322)	13.121	<2 × 10^−16^	***	0.227	0.019	1.255	(1.210, 1.302)	12.106	<2 × 10^−16^	***
Employment	Employed for wages (ref. cat.)	0.000							0.000							0.000						
	Self−employed	−0.539	0.017	0.583	(0.564, 0.603)	−31.471	<2 × 10^−16^	***	−0.463	0.017	0.629	(0.608, 0.651)	−26.805	<2 × 10^−16^	***	−0.498	0.017	0.608	(0.588, 0.628)	−29.488	<2 × 10^−16^	***
	Out of work for >1 year	−0.265	0.028	0.768	(0.727, 0.811)	−9.452	<2 × 10^−16^	***	−0.166	0.029	0.847	(0.801, 0.896)	−5.774	<0.0001	***	−0.221	0.031	0.802	(0.755, 0.852)	−7.120	<0.0001	***
	Out of work for <1 year	−0.256	0.030	0.774	(0.730, 0.822)	−8.472	<2 × 10^−16^	***	−0.145	0.031	0.865	(0.815, 0.919)	−4.728	<0.0001	***	−0.140	0.032	0.870	(0.816, 0.926)	−4.343	<0.0001	***
	A homemaker	−0.164	0.018	0.849	(0.819, 0.880)	−9.007	<2 × 10^−16^	***	−0.139	0.019	0.870	(0.839, 0.903)	−7.443	<0.0001	***	−0.153	0.019	0.859	(0.828, 0.891)	−8.152	<0.0001	***
	A student	−0.042	0.030	0.958	(0.904, 1.016)	−1.429	0.1531	n.s.	0.074	0.030	1.077	(1.016, 1.142)	2.481	0.0131	*	0.030	0.030	1.030	(0.971, 1.094)	0.985	0.3247	n.s.
	Retired	−0.044	0.014	0.957	(0.932, 0.983)	−3.253	0.0011	**	−0.028	0.014	0.972	(0.946, 0.999)	−2.061	0.0393	*	−0.024	0.014	0.976	(0.950, 1.002)	−1.791	0.0733	.
	Unable to work	−0.007	0.018	0.993	(0.957, 1.029)	−0.392	0.6947	n.s.	−0.013	0.019	0.987	(0.951, 1.024)	−0.717	0.4731	n.s	0.005	0.019	1.005	(0.969, 1.043)	0.262	0.7936	n.s.
Health Care Coverage	yes (ref. cat.)	0.000							0.000							0.000						
	no	−0.481	0.017	0.618	(0.598, 0.639)	−28.287	<2 × 10^−16^	***	−0.454	0.017	0.635	(0.614, 0.657)	−26.069	<2 × 10^−16^	***	−0.515	0.020	0.598	(0.575, 0.621)	−25.739	<2 × 10^−16^	***
Personal Doctor(s)	1 Personal Doctor (ref. cat.)	0.000							0.000							0.000						
	2+ Personal Doctors	0.043	0.015	1.044	(1.013, 1.075)	2.838	0.0045	**	0.004	0.015	1.004	(0.974, 1.034)	0.254	0.7996	n.s	−0.013	0.015	0.987	(0.958, 1.017)	−0.840	0.4007	n.s.
	No Personal Doctor	−0.434	0.015	0.648	(0.629, 0.667)	−29.049	<2 × 10^−16^	***	−0.421	0.014	0.657	(0.639, 0.675)	−29.366	<2 × 10^−16^	***	−0.431	0.014	0.650	(0.632, 0.668)	−29.831	<2 × 10^−16^	***
Smoking Status	Every Day (ref. cat.)	0.000							0.000							0.000						
	Some Days	0.093	0.024	1.097	(1.046, 1.151)	3.804	0.0001	***	0.126	0.025	1.134	(1.080, 1.191)	5.057	<0.0001	***	0.068	0.025	1.070	(1.018, 1.124)	2.683	0.0073	**
	Former Smoker	0.313	0.016	1.367	(1.326, 1.410)	20.136	<2 × 10^−16^	***	0.348	0.016	1.416	(1.372, 1.461)	21.796	<2 × 10^−16^	***	0.319	0.016	1.376	(1.333, 1.421)	19.711	<2 × 10^−16^	***
	Never	0.282	0.015	1.325	(1.288, 1.364)	19.269	<2 × 10^−16^	***	0.316	0.015	1.372	(1.332, 1.413)	21.103	<2 × 10^−16^	***	0.316	0.015	1.372	(1.331, 1.413)	20.684	<2 × 10^−16^	***
Annual Household Income	≤$10,000 (ref. cat.)	0.000							0.000							0.000						
	$10,000–$15,000	0.070	0.026	1.073	(1.019, 1.130)	2.659	0.0078	**	0.003	0.027	1.003	(0.951, 1.058)	0.120	0.9047	n.s	0.007	0.028	1.007	(0.953, 1.065)	0.253	0.8005	n.s.
	$15,000–$20,000	0.084	0.025	1.088	(1.035, 1.143)	3.325	0.0009	***	0.053	0.026	1.054	(1.002, 1.109)	2.025	0.0429	*	0.049	0.027	1.050	(0.996, 1.107)	1.822	0.0684	.
	$20,000–$25,000	0.089	0.025	1.093	(1.041, 1.147)	3.596	0.0003	***	0.083	0.025	1.087	(1.034, 1.142)	3.301	0.0010	***	0.043	0.026	1.044	(0.992, 1.099)	1.643	0.1004	n.s.
	$25,000–$35,000	0.154	0.024	1.167	(1.113, 1.224)	6.355	<0.0001	***	0.097	0.025	1.102	(1.050, 1.157)	3.915	0.0001	***	0.076	0.026	1.079	(1.026, 1.135)	2.966	0.0030	**
	$35,000–$50,000	0.130	0.024	1.139	(1.087, 1.194)	5.489	<0.0001	***	0.141	0.024	1.152	(1.098, 1.208)	5.819	<0.0001	***	0.114	0.025	1.120	(1.067, 1.177)	4.531	<0.0001	***
	$50,000–$75,000	0.190	0.024	1.210	(1.154, 1.268)	7.969	<0.0001	***	0.197	0.024	1.218	(1.161, 1.278)	8.093	<0.0001	***	0.157	0.025	1.170	(1.114, 1.229)	6.254	<0.0001	***
	≥$75,000	0.337	0.023	1.401	(1.339, 1.466)	14.527	<2 × 10^−16^	***	0.369	0.024	1.447	(1.381, 1.515)	15.592	<2 × 10^−16^	***	0.312	0.024	1.366	(1.302, 1.432)	12.811	<2 × 10^−16^	***

Signif. codes: 0 ‘***’ 0.001 ‘**’ 0.01 ‘*’ 0.05 ‘.’ 0.1 ‘ ’ 1.
